# 可诱导表达AML1-ETO白血病细胞模型的建立及其对白血病细胞脂肪酸代谢的影响

**DOI:** 10.3760/cma.j.issn.0253-2727.2023.05.003

**Published:** 2023-05

**Authors:** 婉清 谢, 雪 杨, 闰夏 顾, 征 田, 海燕 邢, 克晶 唐, 青 饶, 少伟 邱, 敏 王, 建祥 王

**Affiliations:** 1 中国医学科学院血液病医院（中国医学科学院血液学研究所），实验血液学国家重点实验室，国家血液系统疾病临床医学研究中心，细胞生态海河实验室，天津 300020 State Key Laboratory of Experimental Hematology, National Clinical Research Center for Blood Diseases, Haihe Laboratory of Cell Ecosystem, Institute of Hematology & Blood Diseases Hospital, Chinese Academy of Medical Sciences & Peking Union Medical College, Tianjin 300020, China; 2 天津医学健康研究院，天津 301600 Tianjin Institutes of Health Science, Tianjin 301600, China

**Keywords:** 白血病，髓系，急性, AML1-ETO融合基因, U937细胞, Tet-on系统, 脂肪酸代谢, Leukemia, myeloid, acute, AML1-ETO fusion gene, U937 cells, Tet-on system, Fatty acid metabolism

## Abstract

**目的:**

通过建立可诱导表达AML1-ETO（AE）融合基因的白血病细胞模型，研究AE融合基因对U937白血病细胞生物学功能的影响。

**方法:**

利用慢病毒载体系统，构建强力霉素（Dox）依赖的可诱导表达AE融合基因的U937细胞系（U937-AE）。在AE融合基因表达前后，分别采用CCK-8法、流式细胞术进行细胞增殖、周期、诱导分化检测，并进行转录组学测序和代谢组学测序分析，初步探讨AE融合基因对白血病细胞生物学功能的影响。

**结果:**

①成功构建Dox依赖的Tet-on调控系统，调控AE融合基因在U937-AE细胞中稳定表达。②诱导AE融合基因表达24 h后，表达AE的U937-AE细胞增殖倍率为3.47±0.07，低于AE阴性组的3.86±0.05（*P*<0.05）；处于G_0_/G_1_期的细胞比例为（63.45±3.10）％，明显高于AE阴性组的（41.36±9.56）％（*P*<0.05）；表达CD13、CD14的细胞比例较AE阴性组明显下降（*P*<0.05）。③转录组学测序进行基因集富集分析显示，与静息相关、NF-κB和干扰素α/γ应答相关的炎症反应和免疫调节的基因集被明显富集在表达AE的U937-AE细胞。④U937-AE细胞表达AE融合基因后脂肪酸代谢发生紊乱，AE阳性组细胞的部分中、短链脂肪酸酰基肉碱的代谢物浓度降低［丙酰基-L-肉碱：AE阳性组0.46±0.13，AE阴性组1.00±0.27（*P*<0.05）］；部分长链脂肪酸酰基肉碱的代谢物浓度升高［十四烷酰肉碱：AE阳性组1.26±0.01，AE阴性组1.00±0.05（*P*<0.05）］。

**结论:**

成功建立可诱导表达AE融合基因的白血病细胞模型。AE融合基因表达使U937-AE细胞增殖变慢、周期阻滞、分化受抑，炎症反应和免疫调节的相关基因集被明显富集，细胞的脂肪酸代谢发生紊乱。

## Abstract (en)

**Objective:**

To investigate the effect of the AML1-ETO（AE）fusion gene on the biological function of U937 leukemia cells by establishing a leukemia cell model that induces AE fusion gene expression.

**Methods:**

The doxycycline（Dox）-dependent expression of the AE fusion gene in the U937 cell line（U937-AE）were established using a lentivirus vector system. The Cell Counting Kit 8 methods, including the PI and sidanilide induction, were used to detect cell proliferation, cell cycle-induced differentiation assays, respectively. The effect of the AE fusion gene on the biological function of U937-AE cells was preliminarily explored using transcriptome sequencing and metabonomic sequencing.

**Results:**

①The Dox-dependent Tet-on regulatory system was successfully constructed to regulate the stable AE fusion gene expression in U937-AE cells. ②Cell proliferation slowed down and the cell proliferation rate with AE expression（3.47±0.07）was lower than AE non-expression（3.86 ± 0.05）after inducing the AE fusion gene expression for 24 h（*P*<0.05）. The proportion of cells in the G_0_/G_1_ phase in the cell cycle increased, with AE expression［（63.45±3.10）％）］was higher than AE non-expression［（41.36± 9.56）％］（*P*<0.05）. The proportion of cells expressing CD13 and CD14 decreased with the expression of AE. The AE negative group is significantly higher than the AE positive group（*P*<0.05）. ③The enrichment analysis of the transcriptome sequencing gene set revealed significantly enriched quiescence, nuclear factor kappa-light-chain-enhancer of activated B cells, interferon-α/γ, and other inflammatory response and immune regulation signals after AE expression. ④Disorder of fatty acid metabolism of U937-AE cells occurred under the influence of AE. The concentration of the medium and short-chain fatty acid acylcarnitine metabolites decreased in cells with AE expressing, propionyl L-carnitine, wherein those with AE expression（0.46±0.13）were lower than those with AE non-expression（1.00±0.27）（*P*<0.05）. The metabolite concentration of some long-chain fatty acid acylcarnitine increased in cells with AE expressing tetradecanoyl carnitine, wherein those with AE expression（1.26±0.01）were higher than those with AE non-expression（1.00±0.05）（*P*<0.05）.

**Conclusion:**

This study successfully established a leukemia cell model that can induce AE expression. The AE expression blocked the cell cycle and inhibited cell differentiation. The gene sets related to the inflammatory reactions was significantly enriched in U937-AE cells that express AE, and fatty acid metabolism was disordered.

AML1-ETO（AE）是急性髓系白血病（AML）最常见的融合基因之一，由8号和21号染色体易位形成，主要见于M_2_型AML[1]。AE融合基因能够协同体内发生的其他事件促进AML的发生发展[2]–[4]。与其他类型的AML患者相比，M_2_型预后相对良好，但仍有部分患者最终复发。此外，不具备高强度化疗条件的M_2_型AML老年患者的治疗效果仍然很差[5]，因此更多针对伴AE融合基因AML的预防、治疗措施，需要被不断探索。本研究中，我们构建了强力霉素（Dox）依赖的Tet-on调控系统[6]，调控AE融合基因在U937细胞中表达，并通过体外实验和测序分析方法，初步探讨AE融合基因对白血病细胞生物学功能的影响。

## 材料与方法

一、主要材料、试剂

人白血病细胞系U937细胞由本实验室保存。RPMI 1640、DMEM培养基、胎牛血清（FBS）购于美国Gibco公司；pLVX-Tet-ON Advanced-GFP质粒由本实验室改造构建，psPAX2质粒和pMD.2G质粒购于美国SBI公司；pLVX-Tight-Puro质粒、胶回收试剂盒购于北京TaKaRa生物技术公司；无内毒素质粒中提试剂盒购于北京天根生化科技有限公司；RNA提取试剂盒、逆转录试剂盒、实时定量PCR（qPCR）试剂盒购于北京全式金生物技术有限公司；T4连接酶和限制性内切酶购于英国NEB公司；Dox、嘌呤霉素、Flag单克隆抗体、β-actin小鼠单克隆抗体购于美国Sigma Aldrich公司；乙莫克舍（Etomoxir，E）、西达本胺（Chidamid）、细胞裂解液（RIPA）购于美国MedChemExpress公司；流式细胞术用抗体CD13（PE）、CD14（PE/Cy7）购于美国Biolegend公司；碘化丙啶（PI）、细胞周期检测试剂盒购于上海碧云天生物技术有限公司。

二、细胞培养及诱导表达

1. 细胞培养：人白血病细胞系U937细胞、可诱导表达AE的U937细胞（U937-AE细胞）培养于含10％FBS的RPMI 1640培养基中，人胚肾细胞系HEK-293T培养于含10％FBS的DMEM培养基中。

2. 诱导表达：调整U937-AE细胞密度为2.5×10^5^/ml，按4 ml/孔体系培养于6孔板，加入Dox 500 ng/ml诱导。

三、可诱导表达细胞系U937-AE的建立

实验室前期构建的调控质粒pLVX-Tet-on-Advanced-GFP（rtTA-GFP）和表达质粒pLVX-AML1-ETO-flag（pLVX-AE）分别转染293T细胞进行病毒包装，获取新鲜病毒浓缩后备用，首先用rtTA-GFP病毒感染U937细胞，48 h后经流式细胞仪分选出GFP^+^细胞（U937-rtTA），用pLVX-AE病毒感染稳定表达调控质粒的U937-rtTA细胞，感染48 h后用2.5 mg/ml的嘌呤霉素进行药筛，药筛后再次用流式细胞仪分选出GFP^+^单克隆细胞，经扩增培养后诱导AE融合蛋白表达，即成功构建U937-AE单克隆细胞系。

四、AE表达对U937细胞生物学功能的影响

1. 细胞增殖：设空白对照组（未加Dox诱导，AE阴性）和实验组（加Dox诱导24 h，AE阳性）。在Dox诱导0 h、24 h检测细胞的增殖情况，收集细胞，吹打混匀后取100 ml加入96孔板中，设3个复孔。每孔加入10 ml CCK-8，孵箱继续培养2～4 h，使用Synergy H4 Hybrid Microplate Reader酶标仪，测量450 nm处的吸光度值，计算细胞增殖倍率。实验重复3次。

2. 细胞周期：在Dox诱导0 h、24 h后检测细胞的周期情况，收集细胞，PBS清洗1次弃上清，预冷75％乙醇重悬后轻轻吹打混匀，充分重悬，在4 °C固定至少24 h，固定后的细胞3 000 r/min离心3 min（离心半径11.61 cm），弃上清，加入50 ml PBS的同时加入10 mg/ml Rnase 3 ml，室温孵育10 min，加入PI 150 ml避光10 min，上流式细胞仪检测，Modifit软件分析各细胞周期的比例。实验重复3次。

3. 细胞分化：实验分为空白对照组（Dox−Chidamid−）、Chidamid诱导组（Dox−Chidamid+）、Dox诱导组（Dox+Chidamid−）及Dox序贯Chidamid诱导组（Dox+Chidamid+，用Dox诱导24 h，再用Chidamid处理24 h）。收集细胞，PBS清洗1次弃上清，用200 ml PBS重悬，加入CD13（PE）、CD14（PE/Cy7）抗体各1 ml，4 °C避光孵育30 min，1 ml PBS洗涤1次，200 ml PBS重悬，上流式细胞仪检测。实验重复3次。

五、AE表达对U937细胞转录组学、代谢组学的影响

设置空白对照组（未加Dox诱导，AE阴性）和实验组（加Dox诱导24 h，AE阳性）。实验重复3次。分别收集两组细胞，至少1×10^6^/样本，800 r/min离心5 min（离心半径11.61 cm），弃上清后加1 ml RNAiso Plus，由诺禾致源科技股份有限公司进行RNA测序和代谢组学测序，用GSEA软件分析相关信号通路基因的差异表达，用在线网站（https://www.metaboanalyst.ca/、https://artyomovlab.wustl.edu/shiny/gam/）对代谢组学测序结果进行分析。

六、qPCR实验

设计肉碱棕榈酰转移酶1A（CPT1A）、长链酰基辅酶A合成酶（ACSL1）和酰基辅酶A硫酯酶11（ACOT11）的引物，引物序列见表1。提取空白对照组（未加Dox诱导，AE阴性）和实验组（加Dox诱导24 h，AE阳性）细胞的RNA，逆转录生成cDNA，再进行qPCR。各基因的表达水平采用相对定量法（2^−ΔΔct^）计算，以GAPDH为内参。

**表1:** 实时定量PCR检测不同基因表达水平的引物序列（5′→3′）

基因名称	正向引物	反向引物
CPT1A-2	CAGGCCGAAAACCCATGTTG	CCACCAGTCGCTCACGTAAT
CPT1A-6	CGCTCATGGTGAACAGCAAC	GTGCTGGATGGTGTCTGTCT
ACSL1	AGCCAGAGAAGGCCAAACTC	CCTTTGGGGTTGCCTGTAGT
ACOT11	GTGCCAGAAAGAAGATCCGC	TACAGGCGGACCTGACTGA

七、Etomoxir药物实验

U937-AE细胞在加或不加Dox培养0 h、24 h后再分别给予0、2、4、8 mmol/L Etomoxir作用0 h、24 h，然后检测细胞增殖情况。实验重复3次。

八、Western blot法检测AE融合蛋白表达

U937-AE细胞在加或不加Dox培养24 h、48 h后，收集各组细胞，预冷PBS清洗3遍，计数，每组取1×10^6^个细胞，离心弃上清后加入100 ml RIPA、1 ml PMSF吹打混匀，置于冰上裂解10 min，经超声破碎仪再裂解10个循环，加入25 ml 5×上样缓冲液，置于100 °C金属浴中10 min。配制聚丙烯酰胺分离胶和浓缩胶，每孔加30 µl样品，其中marker加入5 µl，其余用1×上样缓冲液补齐。电泳后转膜至硝酸纤维素膜，将膜浸入含5％脱脂牛奶的PBS-T中，室温封闭 1～2 h，封闭结束PBS-T洗涤3遍，一抗孵育过夜，次日用PBS-T洗涤后加入相应二抗孵育1 h，洗涤后用化学发光成像分析仪照相。

九、统计学处理

使用GraphPadPrism8统计软件对实验数据进行分析；结果以均值±标准差表示，两组之间比较采用非参数*t*检验，*P*<0.05为差异有统计学意义。

## 结果

1. 可诱导表达AE融合基因的U937细胞系的构建及蛋白稳定表达：Dox依赖的Tet-on系统调控AE表达，经药物浓度梯度预实验后选择可稳定表达AE的Dox浓度及处理时间，即500 ng/ml，诱导24 h、48 h，经Western blot法验证，AE融合蛋白均能稳定表达（图1）。

**图1:**
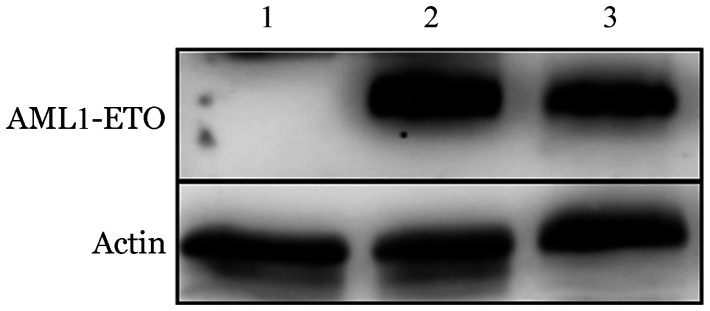
U937-AE细胞中可诱导AML1-ETO融合蛋白表达的验证 **注** 1：空白对照组；2：强力霉素（Dox）作用24 h；3：Dox作用48 h

2. AE融合蛋白表达对U937-AE细胞的生物学功能影响：U937-AE细胞经Dox诱导表达24 h后，细胞增殖倍率为3.47±0.07，明显低于空白对照组的3.86±0.05（*P*<0.05）（图2A）。细胞周期分析结果显示：实验组G_0_/G_1_期细胞比例为（63.45±3.10）％，明显高于空白对照组的（41.36±9.56）％（*P*<0.05）（图2B）。以CD13、CD14作为髓系细胞分化的表面分子标志，观察表达AE后CD13、CD14阳性细胞比例。AE阴性组明显高于AE阳性组（*P*值均<0.05）。AE表达24 h后，再用Chidamid诱导分化24 h，CD13^+^、CD14^+^细胞比例上升（*P*值均<0.05）（图2C），显示AE表达导致U937-AE细胞分化受抑制，但可被Chidamid诱导分化。

**图2:**
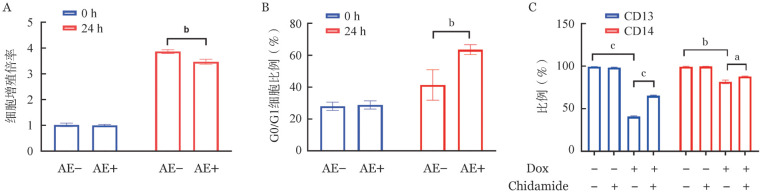
AML1-ETO（AE）融合蛋白表达对U937-AE细胞的生物学功能影响（实验重复3次） A 不同组中细胞增殖倍率；B 不同组中G_0_/G_1_期细胞比例；C Dox、Chidamid不同处理组中表达CD13、CD14分子的细胞比例 **注** Dox：强力霉素；Chidamid：西达本胺。两组比较，^a^*P*<0.05，^b^*P*<0.01，^c^*P*<0.001

3. 对AE阳性、AE阴性细胞进行转录组学测序比较分析：基因集富集分析显示，与静息相关的基因集更多富集在AE阳性组，提示AE阳性细胞表现得更静息（图3A），与此前的细胞生物学功能实验结果一致。NF-κB、干扰素α/γ应答等炎症、免疫调节相关基因集更多富集在AE阳性组（图3B），提示AE阳性细胞的炎症反应、抗凋亡活性增强。

**图3:**
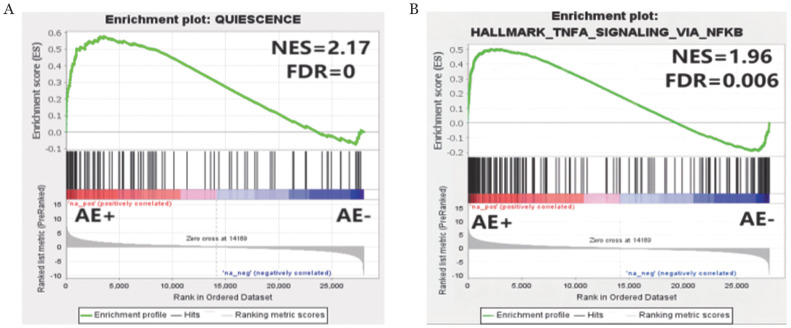
AML1-ETO（AE）阳性、AE阴性细胞转录组学测序部分基因集富集结果 A 静息相关的基因集；B NF-κB基因集

4. 对AE阳性、AE阴性细胞进行代谢组学测序比较分析：转录组学测序基因集富集结果显示，脂肪酸氧化、脂质代谢相关的基因集更多富集在AE阴性组，提示AE阳性细胞的脂肪酸氧化和脂质代谢能力减弱（图4A）。为了进一步了解细胞脂质代谢过程中相关代谢物浓度的差异，进行代谢组学测序，AE阴性组的数据处理后作为基线。结果显示，AE阳性细胞的部分中、短链脂肪酸酰基肉碱的代谢物浓度降低，丙酰基-L-肉碱表达量在AE阳性组为0.46±0.13，明显低于AE阴性组的1.00±0.27（*P*<0.05）；部分长链脂肪酸酰基肉碱的代谢物浓度升高，十四烷酰肉碱表达量在AE阳性组为1.26±0.01，明显高于AE阴性组的1.00±0.05（*P*<0.05）（图4B）。

**图4:**
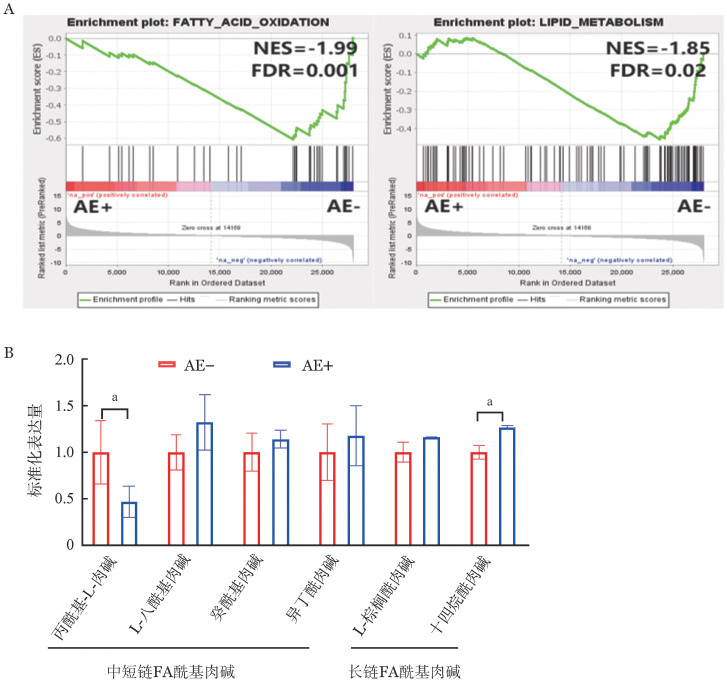
AML1-ETO（AE）阳性、AE阴性细胞转录组学测序部分基因集富集结果及两种细胞代谢组学分析 A 脂肪酸氧化、脂质代谢基因集富集结果；B 中、短链及长链脂肪酸（FA）酰基肉碱的代谢物浓度（实验重复3次） **注** 两组比较，^a^*P*<0.05

5. CPT1A抑制实验：转录组学数据中，AE阳性细胞的CPT1A、ACSL1和ACOT11基因表达低于AE阴性细胞（*P*值均<0.05）（图5A）。通过qPCR检测，再次证实AE阳性细胞的CPT1A、ACSL1、ACOT11基因表达低于AE阴性细胞（*P*值均<0.05）（图5B）。这几种酶是长链脂肪酸代谢的限速酶，利用CPT1A的抑制剂乙莫克舍能够抑制长链脂肪酸的氧化。CPT1A被抑制后，AE阳性和AE阴性细胞增殖均变慢（*P*值均<0.05）（图5C），且呈现剂量依赖性变化，但AE阳性和AE阴性细胞之间差异无统计学意义（*P*值均>0.05）。

**图5:**
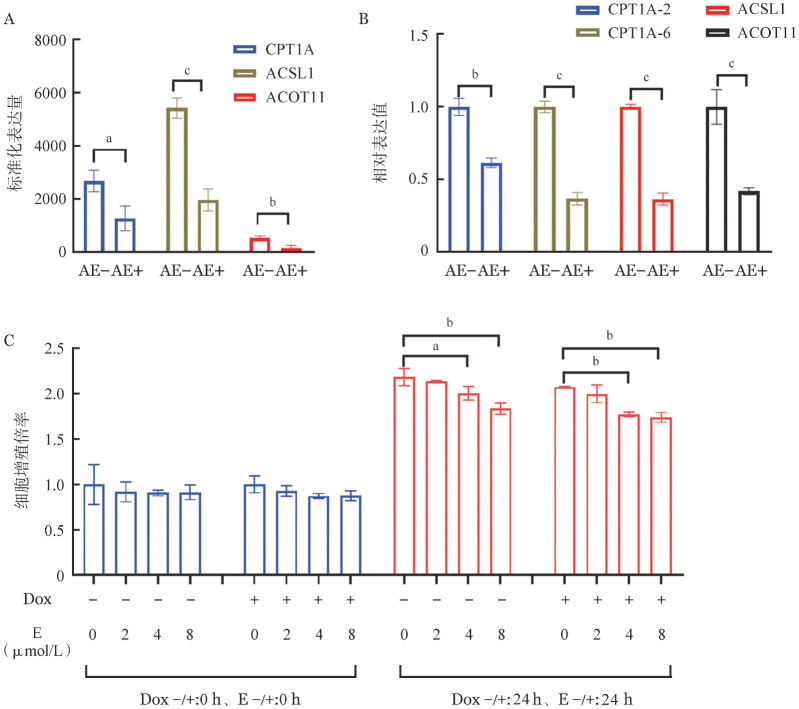
AML1-ETO（AE）阳性细胞中CPT1A的表达变化及抑制CPT1A对细胞增殖的影响（实验重复3次） A 转录组学测序CPT1A、ACSL1、ACOT11表达结果；B 实时定量PCR检测CPT1A、ACSL1、ACOT11表达；C CPT1A抑制实验结果 **注** Dox：强力霉素；Etomoxir（E）：乙莫克舍。两组比较，^a^*P*<0.05，^b^*P*<0.01，^c^*P*<0.001

## 讨论

有多项研究利用不同的方法在U937细胞中表达AE融合蛋白，探索AE在白血病发生发展中的作用[7]–[9]。与AE协同在体内诱导AML的各种事件包括与其结合的蛋白或其调控的基因，研究发现Bcl-2、CEBPA和p14是AE的直接转录靶点[7]，CBFβ是AML1的辅因子，它们之间的相互作用对白血病的发生发展至关重要[10]。

有报道AE的表达使IL-3依赖性L-G细胞的增殖减少了2/3，并诱导Ba/F3小鼠白血病细胞周期的停滞[11]–[12]。AE具有抑制细胞周期和增殖的作用[13]–[14]。本研究中，我们发现AE使U937-AE细胞增殖变慢，细胞周期阻滞于G_0_/G_1_期，推测这中间也存在基因的调节，类似于CBFβ、Bcl-2等与AE的相互作用。白血病细胞表现出周期阻滞是否会在疾病过程中影响治疗效果，需要进一步的药物实验来进行相关的探索。AE通过调节髓系分化中的关键转录因子，包括PU.1和C/EBPβ从而抑制细胞的分化[15]。我们的实验结果显示AE使U937-AE细胞的分化受抑制，但可被Chidamid诱导分化。AE抑制髓系分化不仅驱动白血病发生，还会促使疾病不断进展，这是临床通过诱导分化治疗白血病的基础。

NF-κB是炎症和肿瘤进展的关键调节因子，在白血病发生发展中起重要作用[16]。转录组学测序中我们发现AE表达后，NF-κB信号通路被明显富集。据报道天然AML1通过与IκB激酶复合物相互作用抑制NF-κB信号通路，但AE失去了这种能力，因此，NF-κB信号在表达AE的细胞中被激活[5]，AE表达增强了U937-AE细胞的炎症反应、抗凋亡活性，提示这可能增加了白血病的治疗难度，且在临床工作中可能需要更重视抗炎治疗。

很多因素会导致造血和白血病发生发展过程中代谢重编程的出现，有研究报道Gfi1b缺失后，线粒体底物从葡萄糖转移到脂肪酸，随着细胞从白血病前期发展到白血病，代谢表型也发生了变化[17]。硬脂酸的代谢在健康、白血病前期和白血病人群中存在差异[18]。脂质代谢变化是肿瘤细胞环境中一种重要的改变，可以通过调节转化细胞的增殖、凋亡、迁移和组织侵袭来影响肿瘤的发生、发展和复发[19]–[20]。我们发现AE使U937-AE细胞脂肪酸氧化和脂质代谢能力减弱，CPT1A表达水平下降，脂肪酸代谢发生了紊乱。我们推测脂肪酸代谢不仅在白血病前状态发生改变，而且在白血病发展过程中也发生了改变，AE使U937-AE细胞中脂肪酸代谢紊乱，可能进一步影响了细胞增殖和周期。这些不同的脂肪酸代谢特征可以用来识别白血病前期向白血病进展的过程，以及识别不同白血病人群，从而对不同阶段、不同人群实施相应的干预。CPT1A抑制实验中，AE阴性、AE阳性细胞增殖均变慢，二者之间差异无统计学意义。关于AE如何结合蛋白或调控基因，使白血病疾病过程中呈现不同的脂肪酸代谢特征，以及CPT1A表达水平变化的影响有待进一步研究。
